# Nonverbal Communication in Dementia Family Caregiving: Using the VNVIS-CG Scale for In-Home Video Observations.

**DOI:** 10.1093/geroni/igab046.1850

**Published:** 2021-12-17

**Authors:** Carissa Coleman, Kristine Williams, Kacie Inderhees, Michaela Richardson

**Affiliations:** 1 University of Kansas, Kansas City, Kansas, United States; 2 University of Kansas School of Nursing, Kansas City, Kansas, United States

## Abstract

Communication is fundamental for dementia care and identifying communication behaviors is key to identifying strategies that facilitate or impede communication. To measure caregiver nonverbal communication, we adapted the Verbal and Nonverbal Interaction Scale for Caregivers (VNVIS-CG) for second-by-second behavioral coding of video observations. The VNVIS-CG was adapted for computer-assisted Noldus Observer coding of video interactions captured at home by family caregivers from the FamTechCare clinical trial. Operational definitions for nonverbal communication behaviors were developed and inter-rater reliability was excellent (Kappa = .88) using two independent coders. Videos N=232 were coded featuring 51 dyads; caregivers who were primarily female (80%) spouses (69%) of men (55%) diagnosed with moderate to severe dementia (64.7%). Mean caregiver age was 65 years. Emotional tone conveyed by caregivers was primarily respectful, occurring 68.1% of the time, followed by overly nurturing (9%), bossy, harsh, or antagonistic (6.2%), and silence occurred 16.7 % of the time. Caregiver gestures and positive postures (i.e., animated facial expressions, head nodding, or caregiver body movements) were the most commonly occurring overt behaviors (46.5%), followed by changing the environment to help the PWD (19.9%), and expressing laughter/joy (18.9%). The least common nonverbal behaviors were negative posture, aggression, compassion, and rejecting. The adapted behavioral coding scheme provides a reliable measure that characterizes dementia caregiver nonverbal communication behaviors for analysis of video observations. Ongoing research will identify strategies that facilitate communication as well as determine how strategies vary by dementia stage, diagnosis, and dyad characteristics.

